# Aneurysm in a duplicated internal jugular vein

**DOI:** 10.1093/jscr/rjad366

**Published:** 2023-06-21

**Authors:** Amr Ashour, Dhafer Kamal

**Affiliations:** Department of Surgery, Vascular Surgery Service, Bahrain Defense Forces Hospital, Royal Medical Services, PO Box 28743, Riffa, Bahrain; Department of Surgery, Vascular Surgery Service, Bahrain Defense Forces Hospital, Royal Medical Services, PO Box 28743, Riffa, Bahrain

## Abstract

Internal jugular venous aneurysm (IJVA) is a rare cause of neck swelling that usually remains asymptomatic unless there are complications. We report a case of an aneurysm in a duplicated IJV. Our patient was diagnosed with a palpable soft tissue mass in the neck and was found to have IJVA on imaging. In this case, the duplicated IJV aneurysm was resected, leaving a single internal jugular vein as the main vein draining the ipsilateral head and neck with excellent outcome. The most common indication for surgery is usually cosmetic.

## INTRODUCTION

Aneurysms of the internal jugular veins are usually asymptomatic and are manifested as a palpable soft tissue mass of the neck. They are often misdiagnosed because of the wide range of differential diagnoses that include benign or malignant head and neck neoplasms, such as lymphoma, lymphadenopathy, lipoma, lymphangioma, laryngocele, branchial cyst, cystic hygroma, hemangioma and carotid body tumor [1, 2]. The exact cause remains idiopathic; however, histopathologic findings suggest a localised degenerative process due to an increase in matrix metalloproteinases (MMPs) [3, 4].

## CASE REPORT

**Figure 1:**
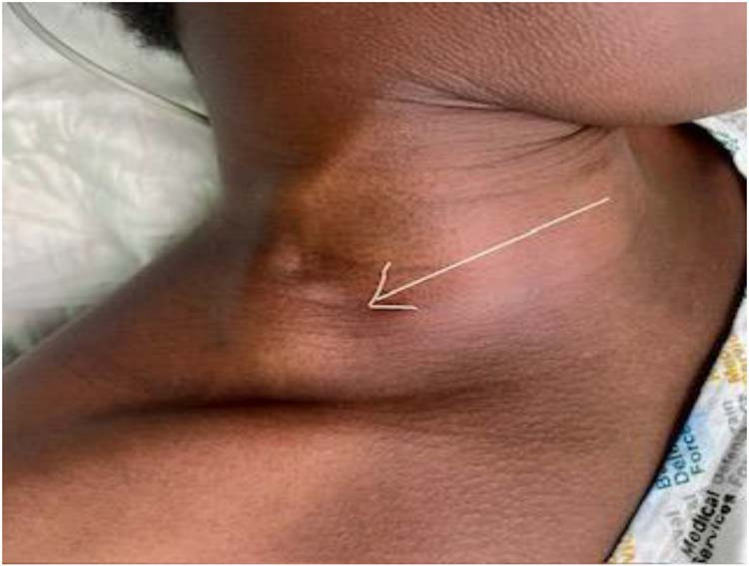
Right-sided neck mass (arrow) that is soft, compressible, non-tender, non-pulsatile, measuring 4 cm × 4 cm and present in the posterior triangle of the neck in the supraclavicular region.

Our case was a 25-y-old female patient from Kenia, East Africa. She has no significant medical or surgical history. She was admitted to our facility complaining of right-sided neck mass recently noticed. On examination, the mass was soft, compressible, non-tender, non-pulsatile measuring 4 cm × 4 cm, present in the posterior triangle of the neck in the supraclavicular region. It appears mainly in supine position and during exercises but disappears when she is upright. A valsalva maneuver caused enlargement of the mass (Fig. 1). The patient’s main complaint was its appearance.

**Figure 2:**
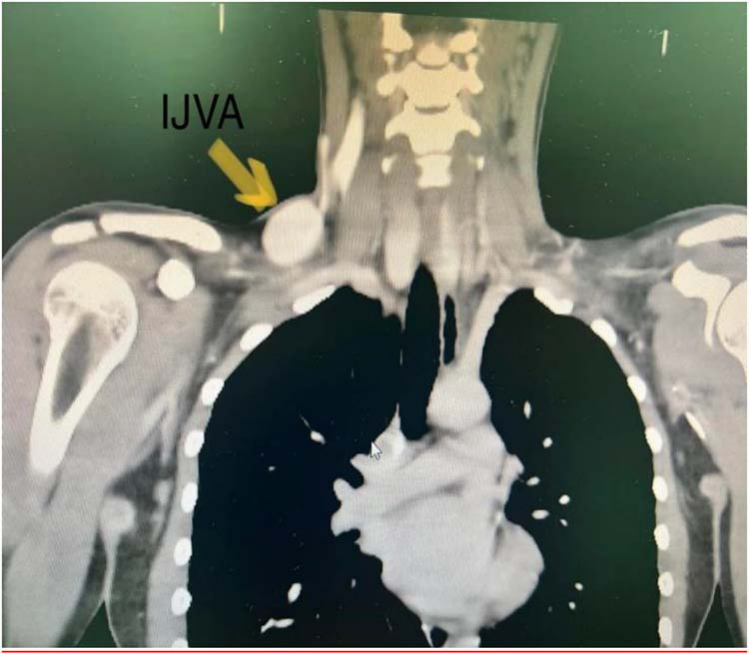
CT with IV contrast showing IJV aneurysm. CT with intravenous contrast showed aneurysmal fusiform venous tributary in right-sided lower neck that originate from right IJV and rejoins IJV before its insertion with the right subclavian vein to form the innominate vein measuring 6 cm in length and 4 cm in maximum diameter. There was no compressing mass in the chest or neck. SVC was normal with no signs of stenosis or compression.

A Doppler ultrasound showed a well-defined, compressible, cystic lesion measuring 5 cm × 2 cm, arising in right supraclavicular region most probably arising from right internal jugular vein. Computed tomography (CT) with intravenous contrast showed aneurysmal fusiform venous tributary in right-sided lower neck that originate from right IJV and rejoins IJV before its insertion with the right subclavian vein to form the innominate vein measuring 6 cm in length and 4 cm in maximum diameter. There was no compressing mass in the chest or neck. SVC was normal with no signs of stenosis or compression (Fig. 2).

**Figure 3:**
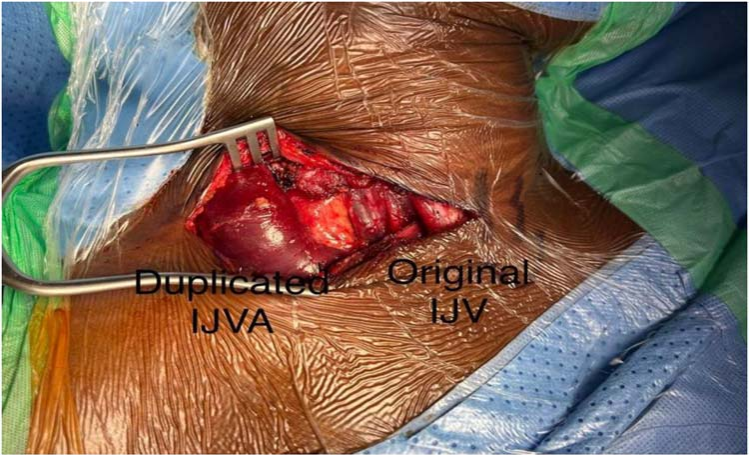
After dissection and identification of duplicated internal jugular vein aneurysm and the original IJV.

The patient’s main concern was the mass appearance. Therefore, she wanted it excised. Consent for surgically excision of internal jugular venous aneurysm (IJVA) was taken. Right supraclavicular transverse incision of the neck was performed, venous aneurysm was identified in posterior triangle of neck (Fig. 3) and was carefully dissected out from its origin from IJV to its insertion in distally in IJV-SCV confluence.

**Figure 4:**
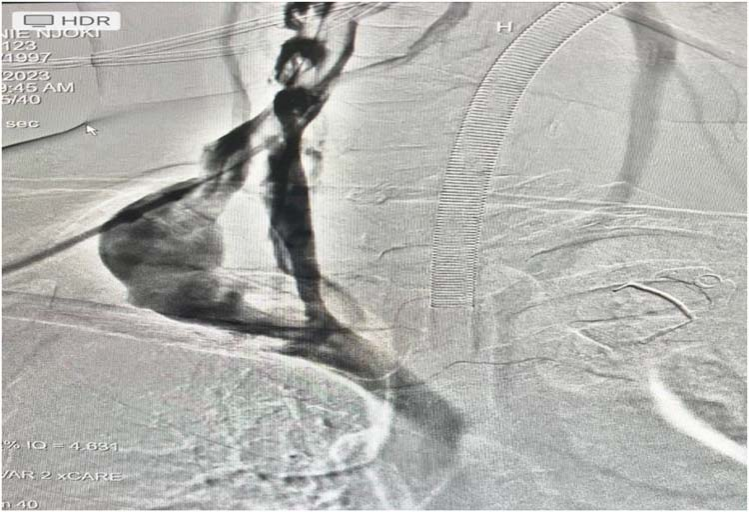
An intraoperative venogram was done through IJV; duplication with an aneurysmal limb is shown.

**Figure 5:**
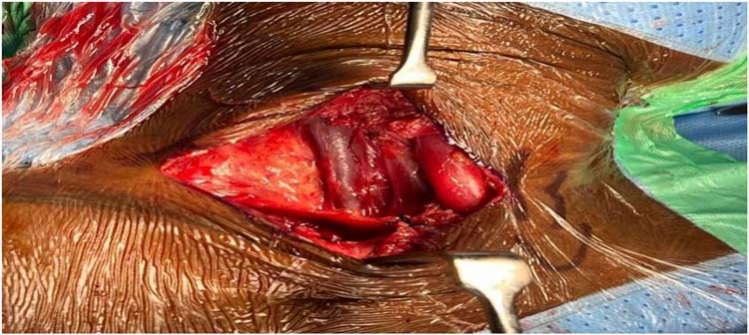
After complete transection of the aneurysm was done.

An intraoperative venogram was done through IJV; duplication with an aneurysmal limb is shown (Fig. 4). Complete transection of the aneurysm was done (Fig. 5).

**Figure 6:**
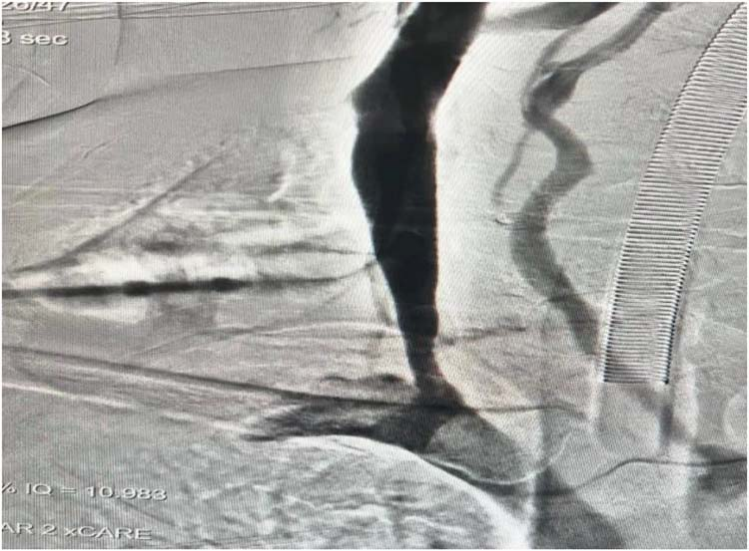
A completion venogram confirmed successful excision of duplicated IJVA.

A completion venogram confirmed successful excision of duplicated IJVA (Fig. 6).

Histopathologic findings suggest a localised degenerative process resulting in thinning of the elastic and muscular layers that confirms the diagnosis of venous aneurysms. The patient did well postoperatively, and he was discharged 1 day later. The patient was happy with the cosmetic result and she gradually resumed activity of daily living. A follow-up neck ultrasound performed 1 month postoperatively showed the patent internal jugular vein with no procedure-related complication in the posterior triangle of the neck.

## DISCUSSION

Aneurysms are abnormal dilatations of the blood vessel wall that may include any parts of the vessel or all its layers [5]. They are more common in arteries, but venous aneurysms have also been described in literature [6]. Venous aneurysms of the neck are rare and could present at any age, with no difference between genders or particular anatomical pattern (e.g. it can be present as a mass in the neck that enlarges with coughing or straining as in our cases) [7]. The majority of patients have asymptomatic mass [8, 9]. Differential diagnoses included hemangiomas, branchial and enterogenous cysts (thyroglossal cyst, dermoid cyst, branchial cyst, cystic hygroma), carotid body tumors, lymphoceles, laryngeal diverticula, cervical adenitis, thyroid mass and persistent jugular lymphatic sac [9, 10]. The lack of guidelines on the treatment of this condition posed a surgical challenge. The diagnosis can be made through ultrasonography, CT with contrast or MRI [9].

The most important complications of venous aneurysms are thrombosis, thrombophlebitis, pulmonary thromboembolism and rupture. Although rare, these risks should be taken into consideration. A recently documented reported of pulmonary thromboembolism caused by an aneurysm of the jugular vein shows that these aneurysms may not be as innocent as previously thought. Therefore, surgical resection of jugular venous aneurysm may be essential to prevent such complications [9–11].

Because of the rare incidence of venous aneurysms, treatment guidelines are not clearly established and the treatment strategies vary [12]. Indication for surgery includes aneurysm complication, pain, swelling and for cosmetic reasons. Moreover, conservative management is the rule in asymptomatic patients [8, 10, 11]. There are anatomical variations of IJV that are present in about 2% of individuals such as duplications, bifurcations, fenestrations, trifurcations and posterior tributary IJV [13–15]. IJV duplication is a rare anatomical variation with an estimated reported prevalence of around 0.4% [13].

This will give the surgeon the opportunity to clearly visualise the anatomic variations and study its course prior to surgery.

This report highlights our surgical approach to a very rare anatomic variation of IJV, which, according to our knowledge, has not been described before; a venous aneurysm in a duplicated IJV who underwent venous aneurysmectomy, leaving the patient with a single normal IJV.

In conclusion, an internal jugular vein aneurysm is a benign condition that can be managed conservatively or surgical resection may be indicated for cosmetic reasons or cases with complications.

## CONFLICT OF INTEREST STATEMENT

None declared.

## FUNDING

This research received no specific grant from any funding agency in the public, commercial, or not-for-profit sectors.
